# Ewing’s Sarcoma of the Breast in a Young Woman: A Case Report and Review of the Literature

**DOI:** 10.3389/fonc.2022.915844

**Published:** 2022-07-12

**Authors:** Simona Papi, Francesca Combi, Silvia Segattini, Silvia Accogli, Enza Palma, Anna Gambini, Alessia Andreotti, Gabriele Luppi, Giovanni Tazzioli

**Affiliations:** ^1^ Division of Breast Surgical Oncology, Department of Medical and Surgical, Maternal-Infantile and Adult Sciences, University Hospital of Modena, Modena, Italy; ^2^ International PhD School in Clinical and Experimental Medicine (CEM), University of Modena and Reggio Emilia, Modena, Italy; ^3^ General Surgery Residency Program, University of Modena and Reggio Emilia, Modena, Italy; ^4^ Division of Oncology, Department of Oncology and Hematology, University Hospital of Modena, Modena, Italy

**Keywords:** breast mass, breast surgery, sarcoma, Ewing’s sarcoma (ES), peripheral primary neuroectodermal tumor, case report, chromosomal translocation

## Abstract

Ewing’s Sarcoma Family Tumors (ESFT) include classic Ewing’s sarcoma of bone, extra-skeletal Ewing’s sarcoma (EES), malignant small cell tumor of the chest wall (Askin tumor), and soft tissue-based Peripheral Primitive Neuroectodermal tumors (pPNET). The t(11;22)(q24;q12) translocation is associated with 85% of tumors and leads to EWS-FLI-1 (Ewing’s Sarcoma–Friend Leukemia Integration-1) formation. This is a potent transforming gene that encodes a chimeric protein that plays a role in the genesis of Ewing’s Sarcoma and Primitive Neuroectodermal Tumors. The breast location of ESFT remains exceptional. The prognosis is among the poorest of all subtypes of breast cancer and even poorer than other extraosseous Ewing’s sarcomas. We describe the case report of a 23-year-old patient with a growing breast lump, who required an accurate and challenging diagnostic estimation and who ultimately resulted in a peripheral primary neuroectodermal tumor (pPNET). Through this case description and a brief narrative review of the literature, we aim to highlight the rarity of ESFT located in the breast. Histopathological confirmation is mandatory for all growing masses of the breast to reach a conclusive diagnosis and plan the correct treatment. Patients with rare diagnoses should always be centralized in breast units, conducting multidisciplinary meetings and, when necessary, the diagnosis should be shared through wider national or international registries.

## Introduction

Ewing’s sarcoma (ES) was first described by Ewing in 1921 (1). Ewing’s Sarcoma Family Tumors (ESFTs) include classic Ewing’s sarcoma of bone, extra-skeletal Ewing’s sarcoma (EES), malignant small cell tumor of the chest wall (Askin tumor), and soft tissue-based Peripheral Primitive Neuroectodermal tumors (pPNET) (2). ES is the third most common primary malignant bone tumor. It occurs more frequently in children and adolescents but is also seen in adults. In white Caucasians >25 years old, ES has an incidence of 0.3 per 100,000 per year, and it is even rarer in the African and Asian populations. The most common ES primary sites are the extremity bones (50% of all cases), followed by the pelvis, ribs, and vertebra. However, any bone is potentially affected and a soft tissue origin is also possible, particularly in adults (30% of cases) (3).

Typical undifferentiated Ewing’s sarcoma is at one end of the spectrum, and pPNET, with clear evidence of neural differentiation, is at the other (4). These are aggressive neoplasms made up of small round monomorphic basophilic cells. However, the specific cell type from which Ewing’s sarcoma takes its origin is still under debate. Once thought to be derived from primitive neuroectodermal cells, many now believe it to arise from mesenchymal stem cells. Expression of the EWS-FLI-1 fusion gene in mesenchymal stem cells changes cell morphology to resemble Ewing’s sarcoma and induces the expression of neuroectodermal markers (5).

Between 20 and 25% of patients are diagnosed with metastatic disease at onset [lung (10%); bone/bone marrow (10%); combination or others (5%)]. With surgery or radiotherapy alone, i.e., without systemic treatments, 5-year survival is <10%. With the currently recommended multimodal approaches including chemotherapy, 5-year survival is around 60–75% in localized and around 20–40% in metastatic disease, respectively, depending on metastatic sites and burden (4). Ewing’s sarcoma family tumors are characterized by chromosomal translocations producing fusion genes that encode aberrant transcription factors. The t(11;22)(q24;q12) translocation is associated with 85% of tumors and leads to EWS-FLI-1 formation (2, 6). In another 10–15% of cases, the translocation t(21;12)(22;12) generates the EWS–ERG fusion, whereas the remaining 1–5% of cases may harbor one of several possible translocations, each resulting in a fusion gene containing a portion of the EWS gene and a member of the ETS (E-twenty-six) family of transcription factors (7). The breast location of ESFT remains exceptional. We describe the case of a 23-year-old woman with a breast lump who was found to have Ewing’s sarcoma. We then performed a brief narrative review of similar cases that were already described in the literature. The review focused on cases in which breast location was the primary tumor site (including metastatic patients at onset), thus excluding all patients in which the breast was a secondary metastatic site.

## Case Description

A 23-year-old woman, with a negative family history of breast and ovarian cancer, presented in December 2020 to the Breast Unit of Modena (Northern Italy) with a growing, palpable mass in the upper-outer quadrant of the left breast.

On physical examination, a 2 cm lump was appreciated, mobile with respect to the underlying planes, and no axillary lymphadenomegalies were found.

Bilateral ultrasounds and mammography revealed a 27 mm solid oval nodule with a markedly inhomogeneous echo structure, absence of calcifications, and moderate reactive axillary lymphadenitis.

A core biopsy showed a monomorphic population of small cells and necrotic areas. Immunostaining was positive for CD 99 and negative for epithelial membrane antigen (MNF 116, CK 7, CK 8, CK 18), p63, synaptophysin, and chromogranin A. Furthermore, immunostaining was negative for lymphoid markers (CD 20/L26, PAX 5, CD 3, CD 4, CD 43, TdT), estrogen receptor, desmin, S-100, and SOX 10. The proliferative activity (Ki-67) was in about 15% of the cells.

The overall picture pointed toward a small cell soft tissue neoplasm. The hypotheses of lymphoproliferative disease and epithelial neoplasia appeared unlikely. A FISH study showed the absence of EWSR 1 translocation. A review of the histological examination of the core biopsy was subsequently performed and judged the findings to be favorable for small round cell sarcoma but more compatible for peripheral primary neuroectodermal tumor (pPNET). Nonetheless, a request for a larger sample of tissue was made to confirm the histological diagnosis.

Taking the diagnostic suspect into account, the patient was subjected to systemic staging with a computed tomographic (CT) scan of the brain, chest, and abdomen and an FDG-PET scan, which ruled out any extramammary origin of the tumor and metastasis.

## Diagnostic Assessment

**Figure 1:**
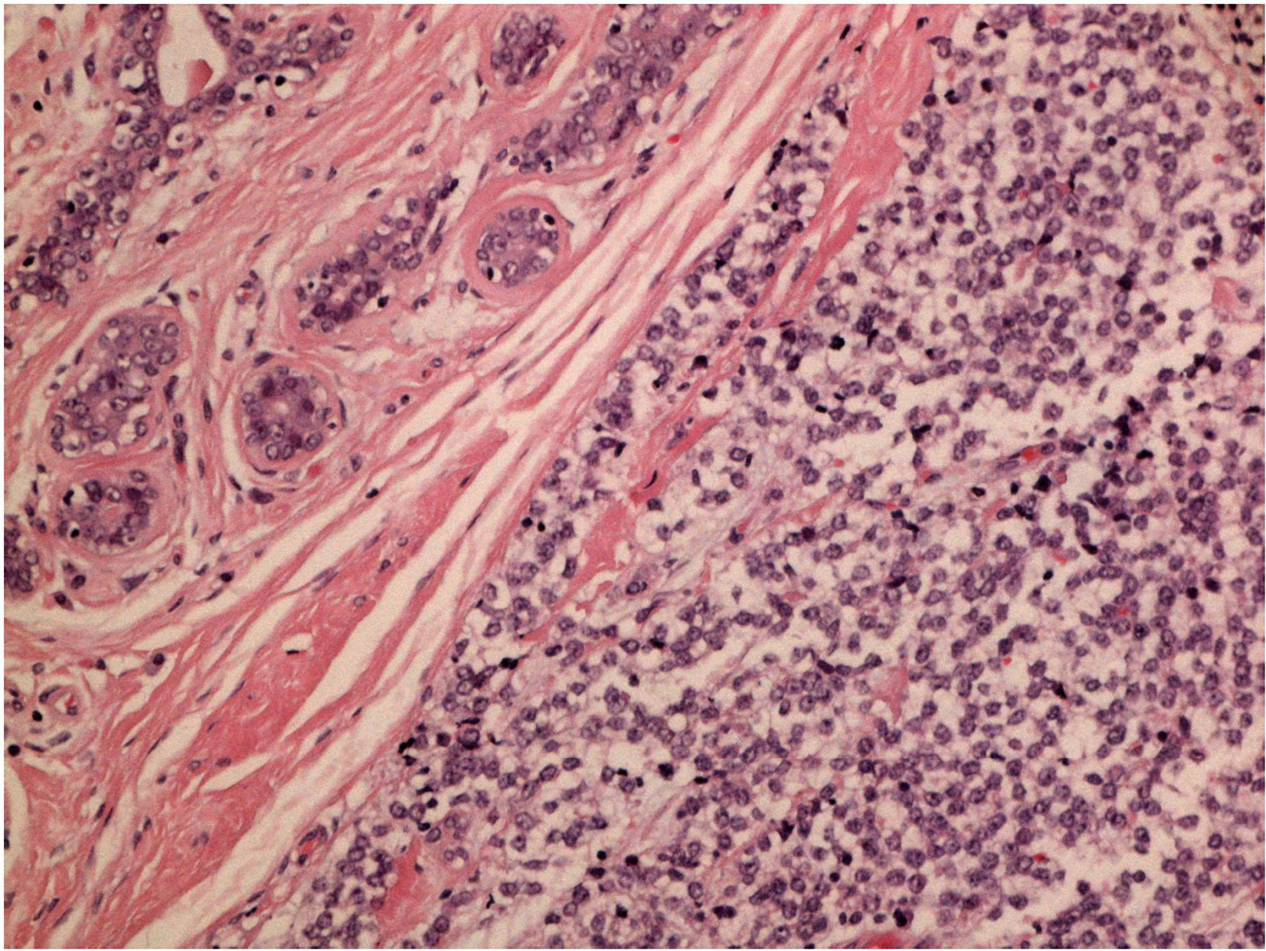
H&E stain 200x magnification.

**Figure 2:**
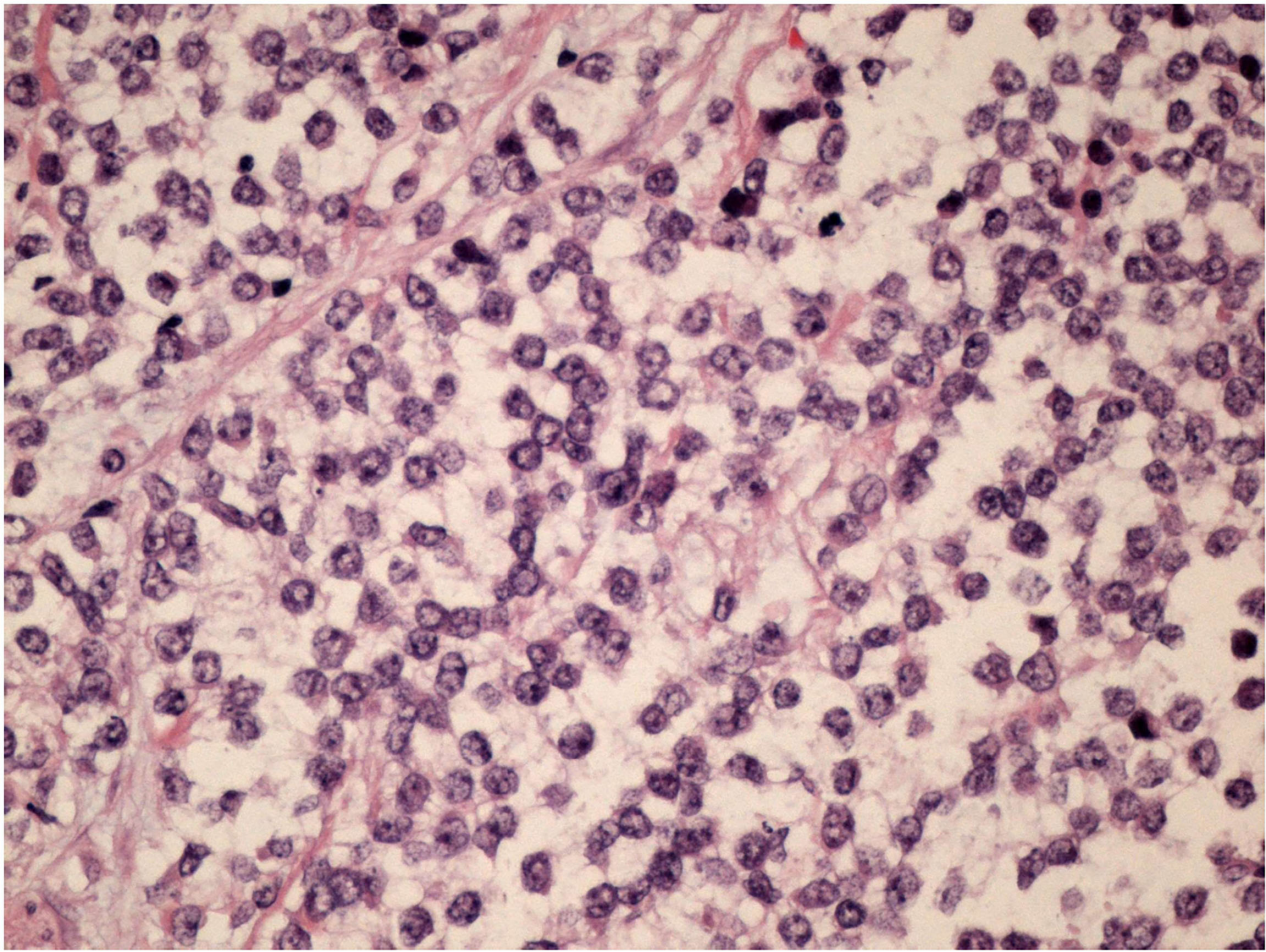
H&E stain 400x magnification.

**Figure 3:**
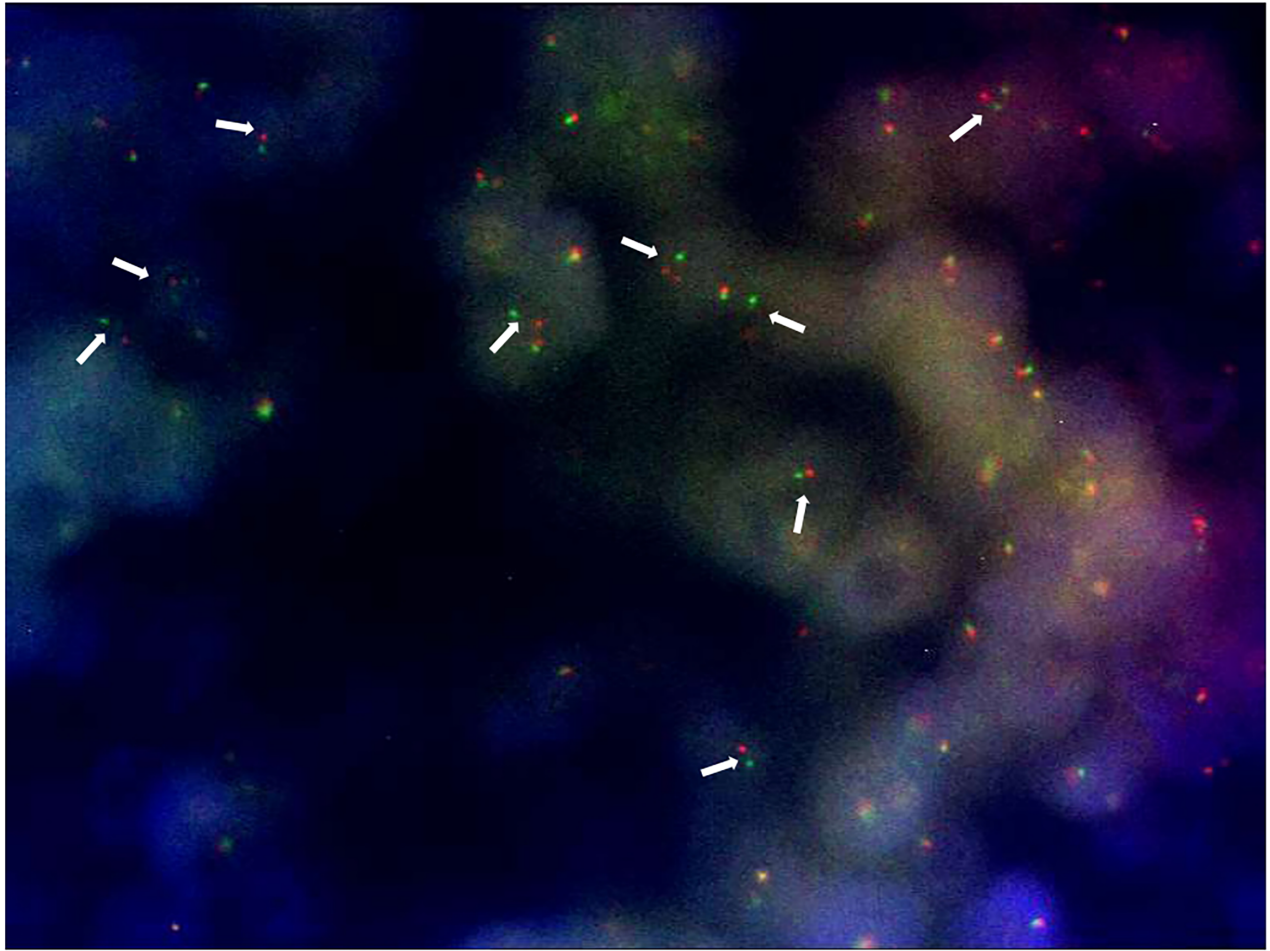
FISH - EWSRI Break Apart FISH Probe Kit (locus 22q12).

After a multidisciplinary discussion, a large lumpectomy was performed for diagnostic purposes. Macroscopically, the specimen of the left breast revealed a tumor measuring 28 mm. The cut surface was brownish gray and friable. Microscopically, the tumor was composed of small round monomorphic cells, with scanty cytoplasm (**Figure 1** *—×*200 magnification and **Figure 2** *—×*400 magnification). Immunostaining was strongly positive for CD 99 and negative for epithelial markers (MNF116, CK 8, CK 18, CK 14), EMA, chromogranin A, synaptophysin, estrogen, and progesterone receptor. Immunostaining was negative for lymphoid markers (CD20/L26, CD 79a, CD 3, CD 10, CD 30/BERH2), desmin, ML actin, CD 34, S 100, and SOX 10. The proliferative activity (Ki-67) was in about 10% of the neoplastic cells. The presence of EWSR 1 translocation was detected through fluorescence *in situ* hybridization (FISH) (**Figure 3**). FKHR translocation was absent. A next-generation sequencing panel (NGS), which detects variants across 52 genes relevant to solid tumors (Oncomine™Focus Assay), was performed and did not show any variants since the specific EWSR1 region is not included. A histological review was also shared with the Italian National Rare Tumors Network, which unanimously confirmed the diagnosis.

Since the tumor reached the superficial margin of the surgical specimen, due to the histological features and small size of the breast, left mastectomy with removal of the nipple–areola complex and subsequent 350 cc breast tissue expander placement, were performed. Histopathological examination revealed no residual tumor in the mastectomy specimen.

Despite the absence of a familial history of breast and ovarian cancer, a genetic consultation was carried out, considering the rarity of the diagnosis in the patient and of the story of an ependymoblastoma at age 30 in the maternal grandmother. A genetic test was performed to search for 22 genes involved in the pathogenesis of hereditary familial tumor syndrome, with the suspicion of an alteration of TP53. No pathogenetic variants were found but only alterations in heterozygosity: c.3787C>T on the gene MSH 6; c.5026A>G and c.7399C>A on the gene APC.

An oocyte pick-up was performed before starting the multi-agent chemotherapy with ifosfamide, vincristine, and adriamycin for 3 cycles. Due to bilateral hand paresthesias, vincristine was replaced with vinblastine for the following 2 cycles. Subsequently, 4 cycles of ifosfamide and etoposide were administered. The GnRH analog was administered throughout the chemotherapy to preserve the ovaries. Follow-up with a total body CT scan is negative after three months of adjuvant chemotherapy and the patient is a candidate to complete reconstructive surgery.

**Figure 4:**
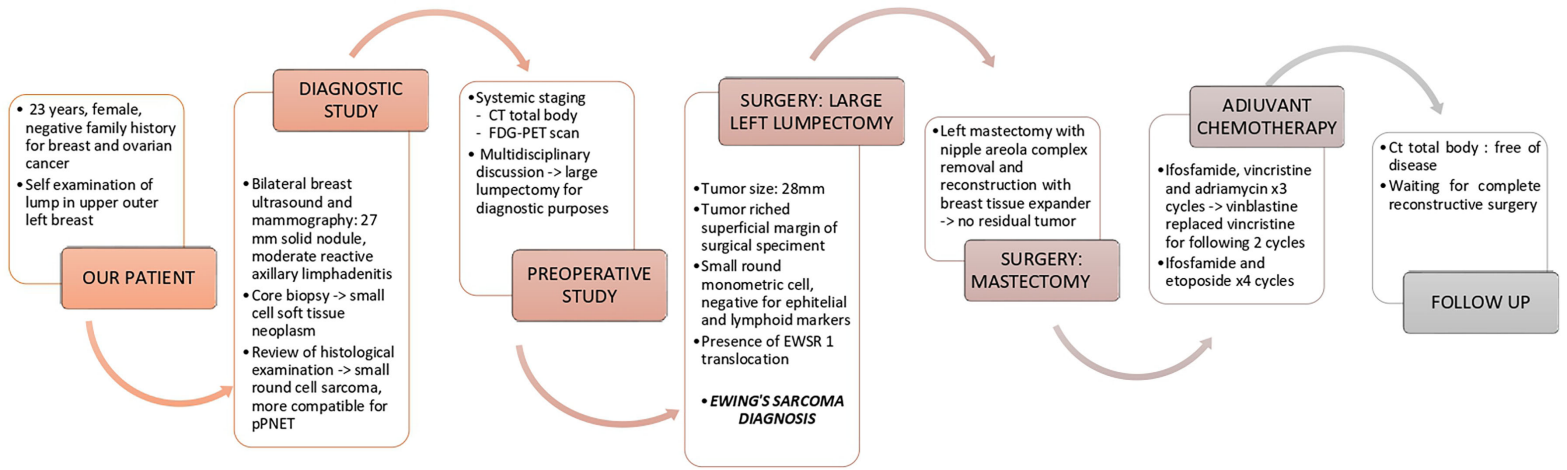
Temporal timeline.

The temporal timeline of relevant diagnostic and clinical milestones is showcased in **Figure 4**.

## Discussion

The breast location of ESFT is an exceptional finding. This clinical entity represents a diagnostic challenge from a clinical, radiological, and histopathological perspective. We described a case of a young woman presenting with a breast lump who was finally diagnosed with Ewing’s sarcoma. We collected a few similar cases that are described in the literature and compared diagnostic assessments and treatment decisions, focusing on patients who presented with breast neoplasm as a primary site of disease and excluding those for whom the breast was a secondary metastasis site, since this aspect strongly affects the choice of correct loco-regional treatment.

The accuracy of breast palpation in evaluating masses is limited because the signs of a malignant lesion are not distinctive. For example, in our patient, the nodule was well delimited and mobile, and the first clinical hypothesis was a fibroadenoma.

Ewing’s sarcoma/pPNET can be studied *via* ultrasonography, computed tomography (CT), or magnetic resonance (MR) imaging. Nonetheless, imaging characteristics are non-specific and they rarely provide a definitive diagnosis. The first level breast imaging (mammography and ultrasound) has features not specific to breast Ewing’s sarcoma/pPNET and they can range from hypoechoic masses with posterior enhancement to heterogeneous masses with areas of necrosis (8). Fluorodeoxyglucose-positron emission tomography (FDG-PET) imaging and computed tomography (CT) scanning are used for detecting metastasis.

The diagnosis of ES/pPNET requires core needle biopsy and pathological examination of the resected sample with immunohistochemistry analysis, and the presence of a t(11;22) translocation allows a definitive diagnosis (9–12). In our case, a FISH study conducted on the core biopsy did not show Ewing’s sarcoma breakpoint region 1 (EWSR1) translocation. Instead, the pathognomonic translocation was detected through FISH after lumpectomy. This suggests that in rare tumors with even rarer localizations, large specimens are often needed to reach a final diagnosis.

The tumor cells are uniformly bland and undifferentiated, with a surprisingly low mitotic index given the rapid growth clinically observed (13). Although our patient clinically presented with a rapidly growing mass, the proliferative activity (Ki-67) of the tumor was 10–15%.

Negativity for epithelial markers ruled out epithelial neoplasia and the absence of lymphoid markers excluded a lymphoproliferative disease.

The aim of our work is precisely to highlight the importance of imaging and pathologic diagnosis of every rapidly growing breast lesion since, although rare, these neoplasms can occur and they can present with unsuspected characteristics.

All patients with palpable breast masses require a thorough family and personal medical history, a careful physical examination, and dedicated radiology. A core needle biopsy or, if not sufficient as in our case, an excisional biopsy, is mandatory in the diagnosis of every rapidly growing breast mass, as confirmed by the clinical cases described in the literature.

**Table 1** summarizes the clinical features and the therapeutic course of primary and metastatic Ewing’s sarcomas/pPNET previously published, all presenting as a breast mass, including our case. In some cases, the mass was initially mistaken for a different type of disease (25), sometimes with a benign lump (8, 15). Maxwell et al. (15) reported a case of a 14 mm breast lump presenting as well delimited and palpable, superficial, oval, isodense, and hypoechoic, that was initially considered for follow-up. The mass was revealed to be a pPNET of the breast on the surgical specimen. Meddeb et al. (8) described a case of a 30 mm lump mistaken at the core needle biopsy with fibrocystic dystrophy. The patient was scheduled to follow up, but the mass increased in a month, up to 130 mm. The lump was removed and the histological examination showed a Ewing’s sarcoma/pPNET. Sahoo et al. discussed the case of a patient who had previously undergone a lumpectomy for a malignant phyllodes tumor. She presented two months after surgery with an 80 mm nodule in the same site, which resulted in a pPNET. Since the treatment is radically different from ductal carcinomas and malignant phyllodes tumors, these rare neoplasms must always be confirmed by immunohistochemistry (25). In an analysis of 21 patients with a mean age of 33 years, with a definitive diagnosis of pPNET/Ewing’s sarcoma presenting with a breast lump, the initial biopsy was misleading on 10 occasions (47.6%) (26), most of which were diagnosed with phyllodes tumor or fibroadenomas. Hence, the importance of centralizing clinical cases in breast units, carrying out multidisciplinary meetings, and consulting more experienced specialists if needed, is recommended every time core biopsy is suspicious or non-diagnostic. In our specific case, only a revision of the first histological examination allowed a diagnosis compatible with pPNET. A subsequent review of the lumpectomy material was performed through the Italian National Rare Tumors Network, which led to a diagnosis of Ewing’s sarcoma.

**Table 1:** Clinical features and therapeutic course of primary and metastatic Ewing's sarcomas/pPNETpreviously published.

Reference	Age at onset	Presentation	Size (cm)	Disease	Treatment	Outcome
**De Silva et al.(**14**)**	35	Breast lump	12 × 7.5	Primary	Chemotherapy+ Radiotherapy	Local and pulmonary recurrence Death after 2 years
**Maxwell et al. (** 15 **)**	35	Breast lump	1.8	Primary	Lumpectomy + Adjuvant chemotherapy	Free of disease after 2.5 years
**Tamura et al. (** 4 **)**	47	Breast lump	2.1 × 1.8	Primary	Mastectomy	N.A
**Popli et al. (**16**)**	14	Breast lump	12	Primary	Wide local excision	N.A.
**Ko et al. (** 17 **)**	33	Breast lump	3 × 2	Primary	Lumpectomy	Free of disease after 6 months
**Dhingra et al. (**18**)**	26	Breast lump	3.5 × 3	Primary	Mastectomy + Adjuvant chemotherapy + Radiotherapy	Free of disease after 1 year
**Vindal and Kakar (**19**)**	26	Breast lump	3 × 2	Primary	Wide local excision+ Adjuvant chemotherapy	Free of disease after 2.5 years
**Suebwong et al. (** 20 **)**	46	Breast lump	4	Primary	Chemotherapy + Radiotherapy	Local and pulmonary progression
**Majid et al. (** 9 **)**	30	Bilateral breast lump	7 (right) 5 (left)	Metastatic	Chemotherapy	Died after 2 cycles of chemotherapy
**Ikhwan et al. (** 21 **)**	33	Breast lump	Locally advanced	Metastatic	Chemotherapy	Died after 3 cycles of chemotherapy
**Meddeb et al. (** 8 **)**	43	Breast lump	13	Primary	Lumpectomy -> Radical mastectomy + Adjuvant chemotherapy	Free of disease after 20 months
**Taşli et al. (** 22 **)**	24	Breast lump	10	Primary	Wide local excision + Adjuvant chemotherapy + Radiotherapy -> local recurrence treated with radical mastectomy	Died 8 months after wide local excision
**Srivastava et al. (** 23 **)**	25	Breast lump	11 × 9 × 6	Primary	Neoadjuvant chemotherapy + Wide local excision	N.A.
**Ranade et al. (** 24 **)**	61	Breast lump and axillary nodal mass	6 × 6 in breast with 5 cm axillary nodal mass	Metastatic	Chemotherapy + Radiotherapy	Died after 2 years
**Our case**	23	Breast lump	2	Primary	Wide local excision -> Mastectomy + Adjuvant chemotherapy	Free of disease after 1 year

The final treatment of Ewing’s sarcoma/pPNET of the breast benefits from surgery, chemotherapy, and radiotherapy (10). Excision margins, larger than those for other breast cancers, are required because they represent the major factor for local control with malignancy. This principle led to performing mastectomy in our patient since the tumor reached the superficial margin in the lumpectomy specimen. ESFT often requires major demolition surgical treatments regardless of their localization. Postoperative radiotherapy plays a role in unresectable disease or when negative margins cannot be obtained (3, 10, 27). After local treatment, systemic chemotherapy improved the 5-year survival rate from 5 to 10% to up to 78 to 87% in previously untreated nonmetastatic Ewing sarcoma (23, 28, 29). Recently, chemotherapic regimens including Vincristine-Topotecan and Cyclophosphamide were shown not to significantly increase survival (29). Chemotherapy also decreases the probability of local recurrence following surgery (10, 30).

Initial large tumor size is a risk factor for a poor prognosis (10, 23, 31) and it is considered the most important prognostic factor in localized disease (10, 17). Also, age ≥18 years is associated with an increased risk of death at 5 years (29).

Metastatic disease at the time of diagnosis is a negative prognostic factor (9, 10, 21) and recurrent Ewing’s sarcoma, both localized and metastatic, is almost always fatal (10, 20, 32). As in soft tissue sarcomas, involvement of the lymph node stations in the axilla is very rare, as they mainly spread by the hematogenous route. Thus, axillary dissection should only be performed in the presence of clinically, radiologically, or histologically proven lymph node disease (33). Kyrillus et al. concluded there was no difference in overall survival (OS) regarding the type of surgery performed (mastectomy versus excision with wide margins) or whether or not lymph node dissection was performed. Patients with tumors <50 mm had a longer overall survival than those with larger tumors (26).

All patients should undergo close follow-up after surgical removal to early diagnose local recurrence and metastasis (19). In this study, the first follow-up was carried out by a complete clinical examination and total body CT scan 3 months after the end of chemotherapy.

Despite the adjuvant treatment, local and pulmonary recurrences are common and the prognosis of pPNET is generally poor (32, 34, 35). The outcome remains among the poorest of all subtypes of breast cancer and even poorer than other extraosseous Ewing’s sarcomas (26).

## Conclusion

We described this clinical case because ES/pPNEt presenting as a breast mass is an extremely rare and uncommon entity, and the diagnosis can often be delayed by misinterpretation.

The knowledge of this possible diagnosis can help with early identification of the disease, early treatment, and an improvement in prognosis.

We focused on emphasizing the importance of centralizing clinical cases in breast units, conducting multidisciplinary meetings, and that histopathological confirmation is mandatory in all growing masses, even if apparently with unsuspicious clinical–radiological characteristics.

## Data Availability Statement

The original contributions presented in the study are included in the article/supplementary material. Further inquiries can be directed to the corresponding author.

## Ethics Statement

Ethical review and approval was not required for the study on human participants in accordance with the local legislation and institutional requirements. The patients/participants provided their written informed consent to participate in this study. Written informed consent was obtained from the individual(s) for the publication of any potentially identifiable images or data included in this article.

## Author Contributions

SP and SA composed the manuscript and literature review. SS provided figures and tables. SS and FC supervised the composition of the manuscript and enriched the discussion. AG, EP, and AA critically revised the work. GT and GL provided the final approval of the version to be published. All authors listed have made a substantial, direct, and intellectual contribution to the work and approved it for publication.

## Conflict of Interest

The authors declare that the research was concluded in the absence of any commercial or financial relationships that could be construed as a potential conflict of interest.

## Publisher’s Note

All claims expressed in this article are solely those of the authors and do not necessarily represent those of their affiliated organizations, or those of the publisher, the editors and the reviewers. Any product that may be evaluated in this article, or claim that may be made by its manufacturer, is not guaranteed or endorsed by the publisher.
